# Direct Cardiac Reprogramming: A Novel Approach for Heart Regeneration

**DOI:** 10.3390/ijms19092629

**Published:** 2018-09-05

**Authors:** Hidenori Tani, Taketaro Sadahiro, Masaki Ieda

**Affiliations:** 1 Department of Cardiology, Keio University School of Medicine, Tokyo 160-8582, Japan; ta.hidenori@gmail.com; 2 Department of Cardiology, Faculty of Medicine, University of Tsukuba, 1-1-1, Tennoudai, Tsukuba City, Ibaraki 305-8575, Japan; taket0901@hotmail.co.jp

**Keywords:** direct reprogramming, cardiomyocytes, cardiac regeneration, gene therapy, fibroblasts

## Abstract

Cardiac diseases are among the most common causes of death globally. Cardiac muscle has limited proliferative capacity, and regenerative therapies are highly in demand as a new treatment strategy. Although pluripotent reprogramming has been developed, it has obstacles, such as a potential risk of tumor formation, poor survival of the transplanted cells, and high cost. We previously reported that fibroblasts can be directly reprogrammed to cardiomyocytes by overexpressing a combination of three cardiac-specific transcription factors (Gata4, Mef2c, Tbx5 (together, GMT)). We and other groups have promoted cardiac reprogramming by the addition of certain miRNAs, cytokines, and epigenetic factors, and unraveled new molecular mechanisms of cardiac reprogramming. More recently, we discovered that Sendai virus (SeV) vector expressing GMT could efficiently and rapidly reprogram fibroblasts into integration-free cardiomyocytes in vitro via robust transgene expression. Gene delivery of SeV-GMT also improves cardiac function and reduces fibrosis after myocardial infarction in mice. Through direct cardiac reprogramming, new cardiomyocytes can be generated and scar tissue reduced to restore cardiac function, and, thus, direct cardiac reprogramming may serve as a powerful strategy for cardiac regeneration. Here, we provide an overview of the previous reports and current challenges in this field.

## 1. Introduction

Cardiac diseases still represent a leading cause of mortality globally, despite decades of development of medical technologies for treating such diseases. Patients with heart disease, such as ischemic heart disease or cardiomyopathy, exhibit increased myocardial fibrosis and loss of cardiomyocytes (CMs), which can progress to severe heart failure, ultimately leading to mortality. As the regulation of fibrosis and inflammation has been thought to be crucial to maintaining cardiac homeostasis, inhibition of the renin–angiotensin system (RAS) using angiotensin-converting enzyme (ACE) inhibitors and angiotensin receptor blockers (ARBs) has been the most validated clinical strategy for treating heart patients. However, these medical therapies have limited effects. A promising approach for severe heart failure patients is heart transplantation; however, the availability of donor organs is very limited. Previous cell-based therapies using bone-marrow-derived cells, mesenchymal stem cells, and cardiac stem/progenitor cells showed low regenerative capacity, mainly due to paracrine signaling, leading to modest effects on cardiac recovery [1,2,3,4,5]. The discovery of induced pluripotent stem cells (iPSCs) has changed the field of regenerative medicine, unveiling a new approach to heart regeneration. iPSCs can theoretically self-renew infinitely and differentiate into various types of cells, including CMs, with high efficiency [6,7]. Moreover, the efficiency of differentiation into CMs has greatly improved with the use of defined small molecules, and metabolic approaches can eliminate iPSCs and have provided a simple method to purify CMs [8,9,10]. However, iPSC-based regenerative medicine needs further refinement as it is associated with obstacles, such as a potential risk of tumor formation, poor survival of the transplanted cells, and the high cost related with large-scale CM production. To overcome such obstacles, we developed a new approach for heart regeneration—direct cardiac reprogramming—in which fibroblasts are converted to induced cardiomyocyte-like cells (iCMs), without first reverting them to stem cells, through the transduction of cardiac-specific factors [11]. Direct cardiac reprogramming in vivo can induce the conversion of resident cardiac fibroblasts (CFs) into functional iCMs in situ and improve cardiac function after myocardial infarction (MI). In this article, we review previous studies on direct cardiac reprogramming and we discuss recent progress made and future perspectives for this novel technology.

## 2. Discovery of Direct Cardiac Reprogramming by Gata4, Mef2c, and Tbx5

**Figure 1:**
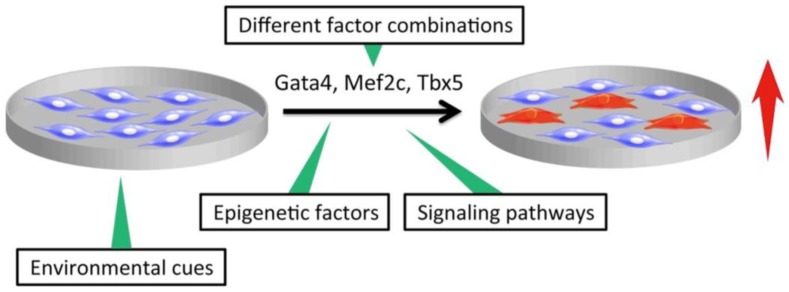
Optimization of transcription factors (TFs), culture conditions, and epigenetic factors to enhance the efficiency of direct cardiac reprogramming.

Davis et al. demonstrated the feasibility of directly converting one cell type into another by overexpressing MyoD to convert mouse fibroblasts into myoblasts in 1987 [12]. In 2006, Takahashi and Yamanaka discovered iPSCs; they showed that somatic cells could be converted into PSCs by the introduction of multiple transcription factors (TFs; Oct4, Sox2, Klf4, and c-Myc). Their innovative discovery inspired a new approach to generate clinically relevant cells by introducing combinations of multiple tissue-specific genes [6,13]. The direct reprogramming of pancreatic exocrine cells into functional β cells by using a specific combination of defined factors was first reported in 2008 by Zhou et al. [14]. We hypothesized that introducing multiple cardiac-specific TFs could directly convert CFs into iCMs. We screened 14 key factors related to cardiac development to find candidate factors for direct cardiac reprogramming [11]. We created CFs from α-myosin heavy chain (αMHC) promoter-driven green fluorescent protein (GFP) transgenic mice (αMHC-GFP), in which mature CMs express GFP, and quantified the reprogramming efficiency by fluorescence-activated cell sorting (FACS) analyses. As a result of the screening, Gata4, Mef2c, and Tbx5 (G, M, and T; GMT) were proved to be sufficient to generate iCMs from neonatal mouse CFs. The iCMs expressed multiple cardiac genes, such as *Myh6* (αMHC), *Actc1* (cardiac α-actin), *Actn2* (α-actinin), and *Nppa* (natriuretic peptide precursor type A), and had well-defined sarcomeric structures. Microarray-based global gene expression analyses revealed that iCMs were similar to neonatal CMs and different from original CFs. The iCMs were found to be epigenetically converted to CMs through analyses of the epigenetic status related to histone modifications and DNA methylation in cardiac-specific gene promoters. The iCMs showed action potentials similar to those of CMs as indicated by electrophysiological analyses. After 4–5 weeks of culture, few iCMs (0.01–0.1% of total cells) showed spontaneous contractions. This suggested that most iCMs remained in a partially reprogrammed or an immature state. Despite this breakthrough in direct cardiac reprogramming, the reprogramming efficiency was very low, and we obtained only few structurally mature CM-like cells. Further studies were aimed at overcoming these problems. Chen et al. demonstrated that the overexpression of GMT through lentiviral transduction was inefficient in converting adult tail-tip fibroblasts (TTFs) and adult CFs into iCMs [15]. The authors showed that GMT-transduced fibroblasts expressed several CM-specific genes and exhibited voltage-dependent calcium currents. However, the transduced cells did not beat spontaneously and were assumed to be functionally immature or partially reprogrammed iCMs. These findings revealed several important points. First, the source cell type and cell conditions are critical for successful reprogramming. Chen et al. used adult TTFs and CFs, while we utilized mainly freshly isolated neonatal CFs, which are epigenetically more plastic [16]. In addition, the culture conditions for fibroblasts and iCMs, including the passage number of the fibroblasts and the serum-containing culture medium, are different between the two studies [11,17]. Second, high expression levels and proper stoichiometry of reprogramming factors are important for reprogramming. GMT expression by lentiviruses might be different from that by retroviruses. Wang et al. generated polycistronic vectors, in which each possible combination of the three factors was constructed in a single transgene. Their findings revealed that two polycistronic vectors, MGT and MTG, which expressed a high level of Mef2c and low levels of Gata4 and Tbx5, enhanced cardiac reprogramming, whereas the other vectors reduced reprogramming efficiency [18]. For successful reprogramming, we should improve reprogramming by optimizing the TFs, culture conditions, and epigenetic factors, as these are important for the induction of iPSCs (Figure 1 and Table 1) [19,20,21].

## 3. Improvement of Direct Cardiac Reprogramming and Cardiac Cell Maturation Efficiencies

### 3.1. Different Factor Combinations

We and other groups have developed and tested multiple cocktails and reprogramming conditions to improve the efficiency of direct cardiac reprogramming and cardiac cell maturation. Protze et al. screened triplet combinations of ten candidate TFs, using a lentiviral system to transduce the genes into mouse embryonic fibroblasts (MEFs) [22]. They analyzed the expression of cardiac genes and demonstrated that the optimal combination for cardiac reprogramming was Mef2c, Tbx5, and myocardin (MTM). They reported that MTM induced cardiac gene expression more strongly than GMT; however, they did not report the generation of functional iCMs. Around the same time, Song et al. investigated the optimal combination of cardiac TFs to generate functional iCMs. They screened six evolutionarily conserved core cardiac TFs in α-MHC-GFP reporter mice. Addition of Hand2 to GMT (GHMT) resulted in more efficient reprogramming of adult CFs and TTFs into functional iCMs compared with GMT alone [23]. This finding suggested that the presence of Hand2 enhanced the efficiency of cardiac reprogramming and cell maturation. We and other groups have analyzed the iCM properties induced by GHMT in more detail [24,25]. Nam et al. reported that 10–30% of GHMT-transduced fibroblasts expressed cTnT, although only a small portion of the cells (~1% of fibroblasts) formed a sarcomere structure that correlated with spontaneous beating activity (~0.16%). Moreover, immunostaining and electrophysiology revealed that GHMT generated immature forms of all three types of myocytes, including atrial, ventricular, and pacemaker cells, in roughly equal proportions. Consistent with this observation, we demonstrated that temporal Hand2 expression improves reprogramming efficiency. To determine the critical timing of Hand2 expression for cardiac reprogramming, we cotransduced retroviral pMX-G, M, T, and doxycycline-inducible lentiviral Hand2, and we found that Hand2 is critical during the first two weeks of cardiac reprogramming. Hand2 promotes cardiac reprogramming at least in part by increasing cardiac marker-expressing cells at the early stage. Hirai et al. found that increasing the transcriptional activity in Mef2c is critical for successful reprogramming for mature iCMs, which is consistent with results obtained using polycistronic vectors, reported by Wang et al. [26,27]. They fused the powerful transactivation domain of MyoD to GHMT and transduced these genes in various combinations into MEFs. Transduction of the chimeric, highly activated Mef2c with the wild-type forms of the other three genes enhanced cardiac reprogramming efficiency. Intriguingly, this approach resulted in the generation of beating clusters of iCMs with an efficiency of 3.5%, which was 15-fold greater than that obtained with wild-type GHMT [26,27].

Addis et al. developed a unique screening system to determine optimal combinations of reprogramming factors to generate functional iCMs. Although many groups have measured reprogramming efficiency by analyzing the expression of cardiac markers or a fluorescent reporter driven by a CM-specific gene promoter, Addis et al. measured quantifiable functions, such as the percentage of cells that show calcium transients. Finally, combination of Hand2, Nkx2.5, Gata4, Mef2c, and Tbx5 (HNGMT) was >50-fold more efficient than GMT alone and produced iCMs with CM marker expression, robust calcium oscillation, and spontaneous beating that persists for weeks following the inactivation of the reprogramming factors [28].

Besides TFs, microRNA (miR) combinations have successfully enhanced reprogramming efficiency. Jayawardena et al. reported that a combination of muscle-specific microRNAs (miR-1, miR-133, miR-208, and miR-499) and a chemical JAK (Janus kinases) inhibitor was capable of inducing iCMs from mouse CFs [29]. Subsequently, we reported that the addition of miR-133a (miR-133) to GMT improved cardiac reprogramming in MEFs, adult mouse CFs, and human fibroblasts [30]. We revealed that the enhancement of direct cardiac reprogramming by miR-133 was mediated via direct repression of Snai1, known as a master regulator of epithelial-to-mesenchymal transition [31]. Intriguingly, overexpression of miR-133 with GMT shortened the time required to induce beating cells from 30 to 10 days compared with GMT alone, suggesting that miR-133 mediates the suppression of Snai1 and the silencing of fibroblast signatures, which favors the transition of fibroblasts toward a CM fate. Thus, this study provided new insights into the molecular mechanisms of cardiac reprogramming; however, maintenance of residual fibroblast identity is the major roadblock during reprogramming toward functional iCMs.

### 3.2. Modification of Signaling Pathways and Environmental Cues

Modifications of signaling pathways and environmental cues have been expected to improve the efficiency of direct cardiac reprogramming and cardiac cell maturation, as these factors are important for the induction of neurons, hematopoietic stem/progenitor cells, and hepatocytes [32,33,34]. Some groups have succeeded in improving reprogramming via the silencing of profibrotic signaling. Ifkovits et al. reported that blockage of the profibrotic signaling pathway by using a transforming growth factor beta (TGF-β) inhibitor, SB431542, enhanced direct cardiac reprogramming of both MEFs and adult CFs to iCMs [35]. Inhibition of TGF-β signaling early in the reprogramming process had the greatest effect. Zhao et al. also inhibited profibrotic signals, such as TGF-β and Rho-associated kinase (ROCK), during reprogramming. Inhibition of these signaling pathways increased the conversion of embryonic fibroblasts into functional iCMs, with an efficiency of ~60% [36]. Conversely, the overactivation of profibrotic signaling attenuated cardiac reprogramming. They compared gene expression in induced and primary CMs and demonstrated that a profibrotic signaling acted as a barrier to cardiac reprogramming by GHMT; reprogramming factor expression is sufficient to activate profibrotic signaling, which must be subsequently suppressed for successful conversion. Subsequently, Mohamed et al. demonstrated that TGF-β and Wnt signaling inhibition greatly enhanced cardiac reprogramming in adult human CFs [37]. They screened 5500 compounds to identify the pathways that can be modulated to enhance reprogramming, and they found that dual signaling inhibition enhanced the efficiency, quality, and speed of direct cardiac reprogramming. Direct cardiac reprogramming in human fibroblasts is further discussed below.

In addition to profibrotic signaling, Zhou et al. screened a library of protein kinases with the aim to modify intracellular signaling pathways. They found that activation of the Akt1 pathway promoted reprogramming efficiency and iCM maturation [38]. Global gene expression of iCMs induced with Akt1 plus GHMT (AGHMT) was shifted toward a state of more mature CMs compared with that of iCMs induced with GHMT. This suggested that Akt1 functions through its downstream targets, activating mTOR and inhibiting Foxo3a, which have important roles in cardiac maturation. Recently, the same group demonstrated that the AGHMT plus zinc finger transcription factor 281 (ZNF281) stimulated cardiac reprogramming in adult fibroblasts [39]. Abad et al. screened seven small-molecule compounds that were previously shown to promote the reprogramming of fibroblasts into iPSCs, and they found that the Notch inhibitor DAPT enhances the efficiency of reprogramming by GHMT [40]. Notch inhibition acts in coordination with Akt1 to increase the acquisition of a mature CM phenotype. The efficiency was up to 70%, with 45% of the reprogrammed iCMs exhibiting spontaneous beating. The authors demonstrated that DAPT increased the binding of Mef2c to the promoter regions of cardiac structural genes to improve cardiac reprogramming efficiency.

We have reported defined culture conditions for efficient cardiac reprograming. Serum-free, defined medium containing several cytokines, fibroblast growth factor (FGF) 2, FGF 10, and vascular endothelial growth factor (VEGF) (FFV) was more appropriate for efficient conversion of fibroblasts into functional iCMs than conventional serum-based culture conditions were [41]. Mechanistically, FFV promoted cardiac reprogramming by activating multiple cardiac TFs, including endogenous Gata4, through the p38 mitogen-activated protein kinase and phosphoinositol 3-kinase/Akt pathways. FFV converted partially reprogrammed cells to fully reprogrammed, functional iCMs by promoting cardiac cell maturation and differentiation at the late stage of cardiac reprogramming (3–4 weeks after induction). Moreover, the addition of IWP4, an inhibitor of Wnt signaling, to FFV-containing medium further increased the generation of beating iCMs by approximately 100-fold compared with conventional FBS-based conditions. Intriguingly, FFV enabled cardiac reprogramming with only two TFs: Mef2c and Tbx5.

Environmental cues, such as extracellular matrix, are also important for cardiac reprogramming. The composition and mechanical properties of the extracellular matrix have been recognized to be important determinants of CM function, and Chopra et al. have demonstrated that the CM cytoskeleton remodels based on substrate stiffness [1,42,43]. Sia et al. improved reprogramming efficiency by combining a microgrooved substrate with an existing optimized culture protocol [44]. Moreover, Li et al. revealed that 3D fibrin hydrogel culture enhances direct cardiac reprogramming [45]. Thus, intracellular signaling pathways and environmental cues not only improve the reprogramming efficiency, but also induce conversion to functionally more mature iCMs.

### 3.3. Epigenetic Factors

During direct reprogramming, CFs must overcome epigenetic barriers to become CMs. To achieve successful reprogramming, reprogramming factors must be able to engage genes that are developmentally silenced and inappropriate for expression in the starting cell population. Transcription factors with “pioneering” activity bind to open the chromatin structure and allow the recruitment of other regulatory factors [46]. Direct reprogramming has proven sufficient to yield a diverse range of cell types from fibroblasts, typically with use of pioneer factors. After Takahashi and Yamanaka discovered iPSC reprogramming factors, other groups revealed that some of these factors including Oct4, Sox2, and Klf4 act as pioneer factors that, together or alone, access closed chromatin [6,47,48]. There are more examples of pioneer factors and co-factors acting in concert to direct cell conversion. In neural direct reprogramming, one of the most advanced research fields in direct programming, the reprogramming factor Ascl1 acts as a pioneer factor by immediately occupying most cognate genomic sites in fibroblasts to open the chromatin structure and allow the recruitment of other neuronal reprogramming factors [49,50]. PU.1 and C/EBPa can reprogram macrophage-like cells from fibroblasts. Intriguingly, C/EBPa also acts as a pioneer factor in iPSC reprogramming [51,52]. Thus, direct cardiac reprogramming necessarily involves changes in the epigenetic state.

In the cardiac field, Gata4 is known as a pioneer factor responsible for driving both cardiac and hepatic lineage conversions [53,54]. Takeuchi et al. achieved ectopic differentiation of embryonic mesoderm into beating cardiomyocytes in mice using transient transfection of Gata4 and the cardiac-specific chromatin-remodeling complex subunit, Baf60c [55]. Recent studies have demonstrated that epigenetic factors can promote the reprogramming process. Liu et al. focused on epigenetic repatterning during direct cardiac reprogramming. They analyzed the levels of trimethylated histone H3 of lysine 4 (H3K4me3) and lysine 27 (H3K27me3), and DNA methylation from day 0 to day 10 of transduction with GMT [56]. A polycistronic vector expressing MGT increased the level of H3K4me3, a marker of transcriptionally active chromatin, and reduced the level of H3K27me3, a marker of transcriptionally inactive chromatin, at the promoter sites of cardiac genes as early as day 3, corresponding with an increase in mRNA expression of the cardiac genes. In contrast, MGT continuously reduced H3K4me3 and increased H3K27me3 at the promoter sites of fibroblast genes until day 10, suggesting that fibroblast genes were progressively suppressed, while cardiac genes were rapidly activated during direct cardiac reprogramming. The authors also demonstrated that the CpG islands at these gene promoters were demethylated as early as day 3, suggesting that DNA methylation contributed to cardiac reprogramming. Dal-Pra et al. also analyzed H3K27 methyltransferase and demethylase expression after transduction with a microRNA cocktail (miR-1, miR-133, miR-208, miR-499) in detail, and their findings confirmed that H3K27 demethylation and derepression of the cardiac transcription factors Tbx5, Mef2c, and Gata4 are crucial for cardiac reprogramming [57]. These findings suggest that reprogramming rapidly activates cardiac gene expression and subsequently represses fibroblast gene expression. Using a loss-of-function screen, Zhou et al. identified Bmi1 as a critical epigenetic inhibitor of reprogramming [58]. Bmi1 is known as a key component of polycomb repressive complex 1, which represses target gene expression through mono-ubiquitination of histone H2A at lysine 119. The authors demonstrated that Bmi1 directly binds the promoter and enhancer regions of several cardiogenic genes including Gata4, and knockdown of Bmi1 promoted an open chromatin status. As a result, the suppression of Bmi1 increased Gata4 expression, leading to cardiac reprogramming with only Mef2c and Tbx5. These findings revealed the importance of epigenetic manipulation and how iCMs are induced in the early stage. Hirai et al. reported that a small-molecule inhibitor of Ezh2, the catalytic component of the PRC2 complex catalyzing H3K27me2/3, increased reprogramming efficiency in the early stage [59]. They also demonstrated that late inhibition of G9a promoted iCM generation, indicating that the timing of histone methyltransferase inhibition is critical to enhancing reprogramming. Recently, Sauls et al. found that chromatin and chromatin-binding proteins were downregulated at 72 h after transduction, indicating that changes in the chromatin state associated with direct cardiac reprogramming start from the early stage [60]. These results suggested that epigenetic changes initiate the orchestrated series of reprogramming steps prior to repression of fibroblast identity.

## 4. Cardiac Reprogramming In Vivo

CFs exist abundantly in the adult heart and proliferate after cardiac injury. Resident CFs can be a potential source of new CMs if they can be directly reprogrammed into functional CMs by direct cardiac reprogramming in vivo. Qian et al. showed that in vivo resident non-CMs were reprogrammed to iCMs by local delivery of GMT after myocardial injury (MI) in mice [61]. They used a retrovirus system to express GMT in infarcted hearts, and performed several lineage-tracing experiments to confirm that resident CFs and non-CMs in the murine heart can be reprogrammed into newly generated iCMs and were not derived from cell fusion with resident CMs. The iCMs had well-formed sarcomeres and functional properties of true CMs. These observations indicated that in vivo reprogramming might yield mature iCMs more efficiently than in vitro conditions. GMT gene transfer improved cardiac functions, including ejection fraction and stroke volume, and scar areas were significantly smaller at 8 weeks after MI in the GMT-treated group than in the control around the same time. Song et al. evaluated in vivo direct reprogramming of proliferating CFs to iCMs by GHMT, using retroviral injection into mouse infarcted hearts [23]. GHMT converted endogenous CFs into functional iCMs in situ and improved cardiac function after MI. Via several lineage-tracing experiments, the authors demonstrated that the iCMs originated from CFs or non-myocytes. Song et al. demonstrated that the ejection fraction and scar size were improved in GHMT-treated mice compared with controls after MI. We have also demonstrated the generation of iCMs in infarcted hearts by GMT retroviral gene transfer in mice [62]. Different from other groups, we generated a polycistronic retrovirus expressing GMT separated by a 2A self-cleaving peptide (3F2A). When we transduced the 3F2A/GFP vector into immunosuppressed mice, the cardiac reprogramming efficiency was approximately 1%. Intriguingly, this polycistronic GMT retrovirus induced morphologically more mature iCMs in fibrotic tissues than the separate GMT vectors, suggesting that polycistronic systems could be valuable tools for in vivo reprogramming. Jayawardena et al. used a lentivirus system to overexpress a miR combo, including miR-1, miR-199, miR-208, and miR-499, and achieved in vivo direct cardiac reprogramming, with improved cardiac function, after MI [63].

**Figure 2:**
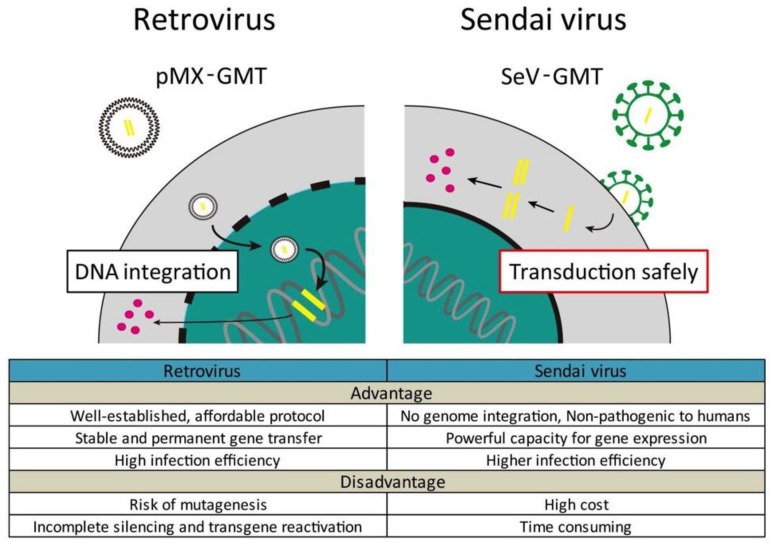
Schematic representation of the different ways of transduction of retrovirus and Sendai virus (SeV) vectors. SeV vectors efficiently and rapidly induce the transformation of fibroblasts into iCMs, without genome integration.

Although we and other groups have demonstrated that gene transfer of appropriate reprogramming factors can improve cardiac function and fibrosis after MI, the use of retroviral and lentiviral vectors leads to insertional mutagenesis. To pave the way to clinical applications, we recently developed a polycistronic Sendai virus vector expressing GMT (SeV-GMT) [64]. SeV vectors have powerful capacity for gene expression and a wide host range, and avoid risks associated with insertional mutagenesis and disruption of gene expression without genome integration (Figure 2) [65]. SeV-GMT efficiently and rapidly reprogramed both mouse and human fibroblasts into iCMs. Moreover, SeV-GMT generated 100-fold more beating iCMs than retroviral GMT and shortened the duration to induce beating cells from 30 to 10 days in MEFs. This new integration-free reprogramming method also promoted the efficiency of cardiac reprogramming in vivo, which resulted in improved cardiac function and reduced fibrosis of mice after MI compared with retroviral injection of GMT. Even though the efficiency of in vivo cardiac reprogramming (~5%) has been lower than that of in vitro reprogramming, in-vivo-generated iCMs are more fully reprogrammed and more closely resemble endogenous CMs than those produced in vitro. This may result from factors within the native microenvironment, such as extracellular matrix, secreted proteins, and tissue stiffness, as discussed above. Although further refinements are needed, cardiac reprogramming with SeV-GMT might be a potential treatment for heart diseases in future.

## 5. Future Directions and Challenges

Direct cardiac reprogramming has undergone great advances and offers several advantages over iPSCs. First, the process is simple and quick; second, it avoids reversion to pluripotency and greatly lowers the risk of contamination of immature cells; and third, direct injection of defined factors avoids cell transplantation [66,67,68,69]. Although the recent progresses in cardiac reprogramming are promising, many hurdles, such as improvement of cardiac reprogramming in human fibroblasts, research in chronic disease models, and understanding the mechanism of cardiac reprogramming and iCM maturation, remain to be overcome.

First, the efficiency of reprogramming of human fibroblasts may still be insufficient to achieve functional cardiac recovery in clinic. We and other groups have reported on the conversion of human fibroblasts into CM-like cells in vitro [70,71,72] (Table 2). Despite some success, direct cardiac reprogramming protocols remain undefined. Compared with murine fibroblasts, it may take extra reprogramming factors and more time to obtain spontaneously beating mature iCMs. Although SeV vectors may be suitable for cardiac reprogramming in human fibroblasts, further studies and refinements are needed [65]. Overcoming issues related to in vivo reprogramming may bring about progress in the fields of gene therapy and regenerative medicine.

Second, experiments in chronic heart failure models are required before clinical application can be contemplated. Although in vivo cardiac reprogramming has allowed impressive improvement of cardiac function and fibrosis, all in vivo studies to date were performed in the acute stage of MI [23,61,62,63]. However, it remains unknown whether in vivo reprogramming could be applied to chronic heart failure models, in which regenerative medicine is in high demand. Moreover, chronic heart failure includes not only ischemic heart disease, but also non-ischemic cardiomyopathy.

Finally, the molecular mechanisms of cardiac reprogramming and iCM functional maturation remain to be clarified. Numerous studies have revealed key players and mechanisms in the early stage of reprogramming, but not in the late stage of reprogramming [30,35,36,57,58]. Recently, Sauls et al. identified temporal global changes in protein abundance during initial phases of iCM reprogramming [59]. The authors observed systematic and temporally distinct alterations in the levels of specific functional classes of proteins, including extracellular matrix proteins, translation factors, and chromatin-binding proteins, during the initial steps of reprogramming. Despite the success of early-stage analyses, it is difficult to acquire sufficient numbers of cells to study the late stage of reprogramming, because of asynchronous, heterogeneous cell populations, and low numbers of fully reprogrammed iCMs. To overcome this limitation, Liu et al. reported a new strategy based on single-cell RNA sequencing [73]. Their study revealed the complexity of the reprogramming process toward iCMs and the important role of a variety of factors such as TFs, non-coding RNAs, cytokines, inhibitors, and epigenetic modifiers. Thus, single-cell transcriptome analysis of the late stage may give us new insights into the mechanism of late-stage reprogramming events and iCM maturation. Moreover, recent studies have revealed that reprogramming factors are intricately functionally connected to reprogramming cocktails, epigenetic factors, and signaling pathways, and these new insights promote advancements in cardiac reprogramming efficiency and maturation [11,39,40]. Thus, integrative studies on the roles of each of the factors discussed in this review are needed to understand the underlying mechanisms.

## 6. Conclusions

Although still in development, direct cardiac reprogramming has undergone great advances and attracted considerable attention. Understanding the molecular mechanism of reprogramming may overcome current technical barriers to promote clinical applications. We strongly believe that, as the demand for heart regeneration is high, direct cardiac reprogramming will advance rapidly, and this new technology can be applied to clinical uses in the near future.

## Figures and Tables

**Table 1:** Cocktails for direct cardiac reprogramming of mouse fibroblasts in vitro. CF: cardiac fibroblast; TTF: tail-tip fibroblast; MEF: mouse embryonic fibroblast.

Different Factor Combinations	Original Cell	Markers and Efficiency
Gata4, Mef2c, Tbx5 (GMT) [11]	CF, TTF	α-MHC+ 20%; cTnT+ 30% of α-MHC; α-Actinin+ most of cTnT+
Gata4, Mef2c, Tbx5 (GMT) [15]	adult TTF, adult CF	α-MHC+ 0%
Puro polycistronic MTG (Mef2c, Tbx5, Gata4) [20]	CF	α-MHC+ 16%; cTnT+ 24%
Mef2c, Tbx5, Myocd [21]	MEF, TTF, CF	cTnT+ 12% (96% of α-MHC)
Gata4, Hand 2, Mef2c, Tbx5 (GHMT) [22]	adult TTF, CF	αMHC+ cTnT+ 9.2% (TTF), 7.5% (CF)
Chimeric Mef2c + GHT [26]	MEF	cTnT+ 20.9%; beating iCMs 3.5%
Hand2, Nxk2.5, Gata4, Mef2c, Tbx5 (HNGMT) [28]	MEF, CF	GCaMP5 activity+ 1.6%
MiR-1, miR-133, miR-208, miR-499 [27]	CF	αMHC+ 1.5% to 7.7%
GMT + miR-133 [30]	MEF, CF	αMHC+ 19.4%; cTnT+ 5.4%
**Signaling Pathways**		
MicroRNA cocktail + JAK inhibitor I [29]	CF	αMHC+ ~28%
HNGMT + TGFβ inhibitor [35]	MEF, adult CF	cTnT-GCaMP5+ activity: 16.95%
GHMT + ROCK/TGFβ inhibitor [36]	MEF, adult CF	cTnT+ ~67%; α-actinin+ ~64%
GMT + FGF2, FGF10, VEGF [41]	MEF, TTF	αMHC+ 15%; cTnT+ 5%
GMT + WNT/TGFβ inhibitor [37]	CF	αMHC+ 38.3%; cTnT+ 23.4%; cTnT-GCaMP5+ activity 12.5%
GHMT + Akt1 (AGHMT) + Notch inhibitor [40]	MEF	cTnT+ 70%; Ca flux+ 40%; beating 45%
GHMT + Akt1 (AGHMT) + ZNF281 [39]	TTF	αMHC+ ~33%; cTnT+ ~45%
**Environmental Cues**		
GMT in microgrooved substrate [44]	TTF	αMHC+ 32%; beating more than double
**Epigenetic Factors**		
MGT + Bmi1 [58]	CF	αMHC+ 30%; cTnT+ 28%

**Table 2:** Cocktails for direct cardiac reprogramming of human fibroblasts in vitro.

Different Factor Combinations	Original Cell	Markers and Efficiency
Gata4, Hand2, Tbx5, Myocd, miR-1, miR-133 [70]	nHFF, DF, CF	cTnT ~19% (nHFF); calcium transient+
Gata4, Mef2c, Tbx5 (GMT) + Mesp1, Myocd [71]	DF, CF	cTnT 5% (CF); sarcomeric structure+
GMT + Esrrg, Mesp1, Myocd, ZFPM2 (7F) [72]	hESC-derived fibroblast	cTnT 13%; Ca flux+, action potential
GMT + Mesp1, Myocd, miR-133 [30]	CF	cTnT 23–27%
GMT + Myocd, WNT/TGFβ inhibitor [37]	CF	cTnT >2-fold compared with 7F; Calcium transients ~80%
SeV-GMT [64]	CF	cTnT 15%; Synchronous with surroundings by cocultivation

nHFF: neonatal human foreskin fibroblast; DF: dermal fibroblast; CF: cardiac fibroblast.
